# The cerebrovascular and neurological impact of chronic smoking on post-traumatic brain injury outcome and recovery: an in vivo study

**DOI:** 10.1186/s12974-020-01818-0

**Published:** 2020-04-27

**Authors:** Farzane Sivandzade, Faleh Alqahtani, Ali Sifat, Luca Cucullo

**Affiliations:** 1 Department of Pharmaceutical Sciences, Texas Tech University Health Sciences Center | Jerry H. Hodge School of pharmacy, 1300 S. Coulter Street, Amarillo, TX 79106 USA; 2 Department of Pharmacology and Toxicology, College of Pharmacy, King Saud University, Riyadh, 11451 Saudi Arabia; 3 Center for Blood-Brain Barrier Research, Texas Tech University Health Sciences Center, Amarillo, TX 79106 USA

**Keywords:** Tobacco smoke, Trauma, Brain injury, Oxidative stress, Blood-brain barrier, Tight junctions, Nrf2, Antioxidant

## Abstract

**Background:**

Traumatic brain injury (TBI) is among the most prevalent causes of cerebrovascular and neurological damage worldwide. To this end, tobacco smoke (TS) has been shown to promote vascular inflammation, neurovascular impairments, and risk of cerebrovascular and neurological disorders through oxidative stress (OS) stimuli targeting the blood-brain barrier (BBB) endothelium among others. It has been recently suggested that premorbid conditions such as TS may exacerbate post-TBI brain damage and impact recovery.

**Methods:**

Our study investigated the mechanisms underlying the exacerbation of TBI injury by TS using a weight drop model. For this purpose, male C57BL/6J mice, age range 6–8 weeks, were chronically exposed to premorbid TS for 3 weeks. Test animals were then subjected to TBI by guided vertical head weight drop using a 30 g metal weight free felling from an 80 cm distance before reaching the target. We analyzed the physical activity and body weight of the mice before TBI and 1 h, 24 h, and 72 h post-injury. Finally, mice were sacrificed to collect blood and brain samples for subsequent biochemical and molecular analysis. Western blotting was applied to assess the expression of Nrf2 (a critical antioxidant transcription factor) as well as tight junction proteins associated with BBB integrity including ZO-1, Occludin, and Claudin-5 from brain tissues homogenates. Levels of NF-kB (a pro-inflammatory transcript factor which antagonizes Nrf2 activity) and pro-inflammatory cytokines IL-6, IL-10, and TNF-α were assessed in blood samples.

**Results:**

Our data revealed that premorbid TS promoted significantly increased inflammation and loss of BBB integrity in TBI when compared to TS-Free test mice. Additionally, mice chronically exposed to TS before TBI experienced a more significant weight loss, behavioral and motor activity deficiency, and slower post-TBI recovery when compared to TS-free TBI mice.

**Conclusion:**

The effects of premorbid TS appear consequential to the abrogation of physiological antioxidative and anti-inflammatory response to TBI leading to worsening impairments of the BBB, OS damage, and inflammation. These factors are also likely responsible for the retardation of post-traumatic recovery observed in these animals.

## Background

Traumatic brain injury (TBI) has long been among the most common type of trauma and the leading cause of death and disability in the young-aged population (≤ 45 years of age) in the USA, thus becoming a serious public health concern in modern society [1, 2]. According to the Centers for Disease Control and Prevention (CDC), every year in the USA, about 2.5 million people seek emergency care for TBI injuries secondary to motor vehicle accidents, falls, assaults, sports-related events, and other mechanisms. Currently, more than 5.3 million Americans are living with a lifelong disability due to TBI [3]. The effects of TBI can cause emotional, physiological, cognitive, motor, and behavioral damage ranging from mild to severe deficits and death [4–7]. Mild traumatic brain injury (mTBI) accounts for over 80% of head injuries [3, 8]. mTBI typically results in transient symptoms, including sensitivity to light and sound, headache, vision impairment, difficulties with cognition, and balance.

The pathophysiology of TBI can be divided into primary and secondary injury mechanisms. The primary mechanical injury is due to the physical harm and may result in intracranial or extracranial hemorrhage following damage to the blood vessels, brain tissue, and the blood-brain barrier (BBB) [9]. The secondary injury occurs within days, weeks, months, or even years after the first injury and derived from oxidative stress, inflammation, imbalanced calcium homeostasis, excitotoxicity, apoptosis, increased vascular permeability, and BBB disruption [2, 10–12]. Although the initial brain injury is the main pathogenic factor, secondary brain injury is generally more severe and complex than the primary one. It encompasses anatomical, cellular, molecular, and behavioral changes [12–15]. A series of delayed secondary biochemical and metabolic changes at the cellular level is prodromal to other pathological processes, including oxidative stress, inflammation, excitotoxicity, enhanced vascular permeability, and BBB impairment resulting in exacerbated post-traumatic brain damage and eventual neuronal dysfunction [2, 15, 16]. Post-traumatic dysfunction of the BBB is one of the significant factors determining the progression of injury and affecting the time course and the extent of neuronal repair [17]. There is now a wealth of evidence suggesting the detrimental role of oxidative stress in the dysfunction of BBB in the cerebrovascular level [18]. It is well established that reactive oxygen species (ROS) causes DNA damage by introducing single- and double-stranded DNA breaks leading to delayed neurobehavioral recovery post-TBI [19, 20].

Moreover, disrupted tight junctional proteins (including occludin and claudins) reduce BBB integrity, thus increasing paracellular permeability. ROS is an active pro-inflammatory promoter which impacts endothelial viability and vascular biological responses. These latter include upregulation cell adhesion molecules, inflammatory cytokines, and, eventually, the influx of inflammatory cells into the brain parenchyma. These factors ultimately determine the progression of injury, including excitotoxicity and neuronal loss.

ROS constituents and other reactive compounds profoundly enrich tobacco smoke (TS). Chronic smoking has been clearly shown to promote dysfunction of the BBB through activation of oxidative, inflammatory, and immune responses that support the onset and progression of cerebrovascular and neurodegenerative disorders, including TBI [21–26]. Therefore, it is not surprising that chronic smoking is one of the most prevalent premorbid factors likely to influence the severity of TBI and retardation of post-TBI recovery. This data may explain why TBI patients with premorbid TS exposure exhibit aggravated post-traumatic cerebrovascular inflammatory and neurovascular conditions when compared to non-smokers [3]. TS will likely add to post-traumatic oxidative stress further worsening peroxidation of membrane polyunsaturated fatty acids, protein carbonylation, and DNA oxidation through ROS generation. All these pathogenic processes ultimately affect the BBB permeability and fluidity leading to membrane damage and eventual apoptosis and tissue necrosis [20, 27, 28]. Furthermore, it is well known that nicotine in cigarettes also causes activation of nicotine receptors leading to acetylcholine-reliant liberation of nitric oxide from vascular endothelium, which in turn leads to increased permeability of BBB, resulting in loss of brain homeostasis. The nicotine also negatively affects Na/k/2Cl- co-transporter present on the luminal surface of BBB and causes blood thickening, which impairs blood flow.

The well-known association between smoking and vascular endothelial dysfunction, as well as the increased risk of neurological disorders (such as stroke, vascular dementia, small vessel ischemic disease, multiple sclerosis, Alzheimer’s, etc.), is given. Despite the epidemiological and translational studies strongly suggesting activation of pathophysiological pathways by TS that exacerbate TBI outcome and influence recovery, determination and characterization of shared key modulators in BBB impairment due to TS and TBI lies unexplored. Identification and then targeting of these putative key modulators could help in preventing the initiation of metabolic/cerebrovascular complications due to TBI in smokers. Thus, the goal of the present study was to characterize the pathogenic impact of chronic smoking on TBI and assess the post-traumatic exacerbation of TBI, by studying key established pathological parameters leading to loss of BBB function and integrity. For this purpose, we have used a well-established weight-drop TBI mice model, to test the impact of premorbid TS exposure on post TBI injury and recovery animals with or without chronic pre-exposure to TS. We believe that a better understanding of TS potential influence on TBI can facilitate the development of more targeted and effective interventions to improve post-traumatic outcomes as well as reducing the impact of chronic smoking itself. This latter is of paramount importance for patients that cannot quit smoking. Furthermore, subjects who have recently stopped smoking remain at high risk of cerebrovascular disorders and worse secondary brain injury outcomes (due to the lingering effect of chronic smoking) for a relatively long period.

## Methods

### Materials and reagents

Reagents and chemicals were purchased from Sigma-Aldrich (St. Louis, MO, USA) or Bio-Rad Laboratories (Hercules, CA, USA). All Quantikine ELISA kits were purchased from R & D systems. The antibodies used in this study were obtained from various sources: rabbit anti-ZO-1 (#402200), mouse anti-Occludin (#331500), and anti-Claudin-5 (#4C3C2) from Life Technologies; mouse anti-PECAM-1 (#sc-376764), mouse anti-VCAM-1 (#sc-13160), rabbit anti-Nrf2 (#sc-722), mouse anti-NQO-1 (#sc-376023), mouse anti-HO1 (#sc-390991), and mouse anti-NFκB-p65 (#sc-(F-6)-8008) from Santa Cruz Biotechnology. Donkey anti-rabbit (#NA934) and sheep anti-mouse (#NA931) HRP-linked secondary antibodies were obtained from GE Healthcare (Piscataway, NJ, USA).

### In vivo experimental design

**Fig. 1:**
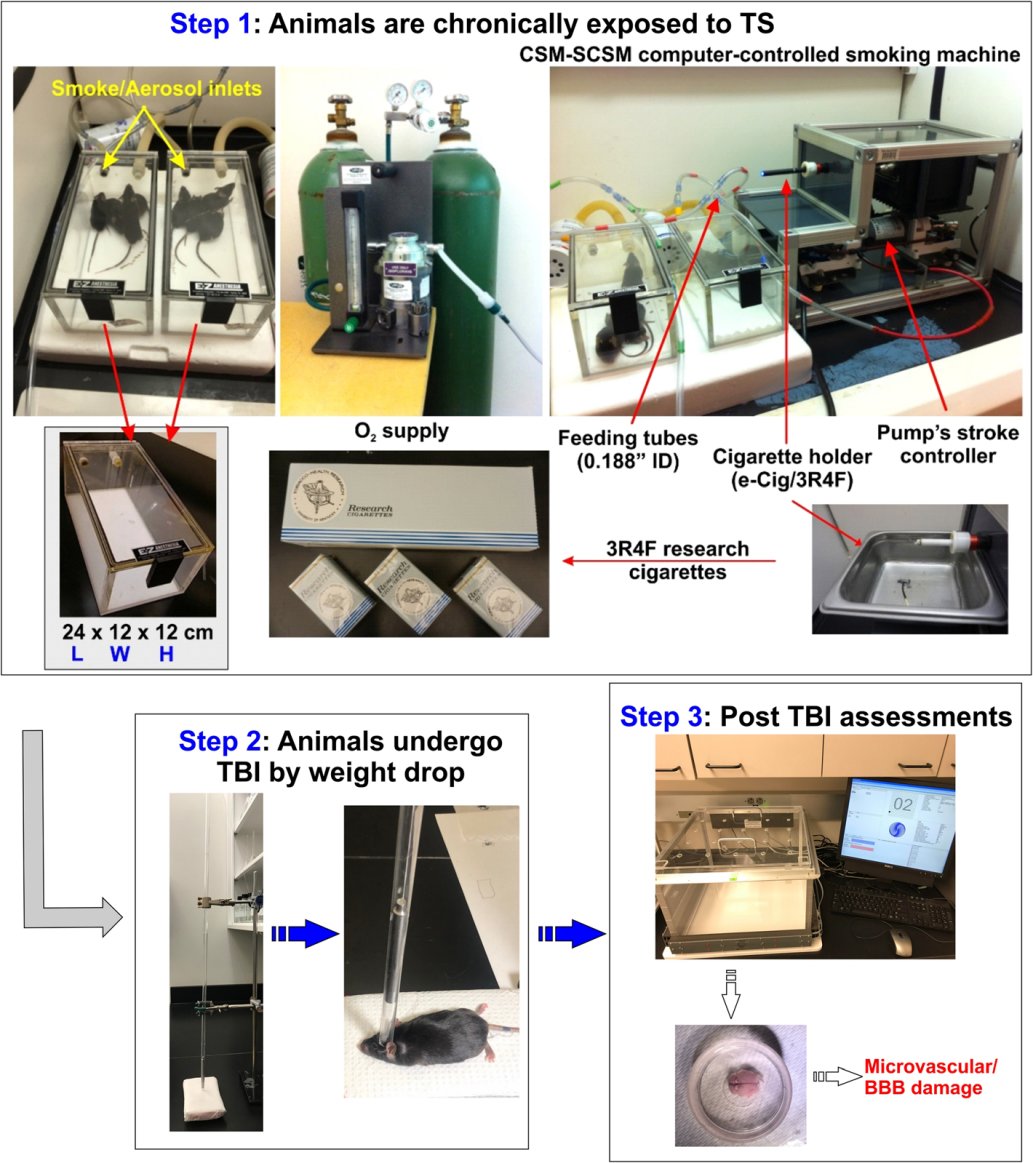
Experimental flow and set up including cigarette smoke generation, animal exposure, induction of traumatic brain injury, and post-traumatic assessment of motor activity. C57BL/6J mice were chronically exposed to TS (full body side stream exposure) for 3 weeks using a computer-controlled single cigarette smoking machine using the FTC approved smoking protocol. Test animals were subjected to TBI by head weight (30 g) drop from an 80-cm height through a pre-positioned vertical guide. Physical activity and weight of the mice were analyzed before TBI and at 1 h, 24 h, and 72 h after TBI using an open-field test. Finally, mice were sacrificed to collect blood and brain samples for subsequent biochemical and molecular analysis

The experiment protocol in this study was abided by the Institutional Animal Care and Use Committee, TTUHSC, Lubbock, Texas [29]. C57BL/6J male mice (ranging between 6 and 8 weeks old and a bodyweight comprised between 20 and 22 g) were purchased from Jackson Laboratory. Mice were divided into four major groups (six animals/group), including control, TBI, smoke (TS-exposed animals), and smoke + TB. Animals were given 3 days for acclimatization post-arrival in the new location to recovery from the transport. All mice were given unlimited access to standard mouse chow and water. Of these mice, the smoke only and smoke + TBI test groups were chronically and simultaneously exposed (via direct inhalation) to sidestream smoke derived from 3R4F standardized research cigarettes (9.4 mg tar and 0.726 mg nicotine/cigarette equivalent to full flavor commercial products). Sidestream smoke was generated using a single cigarette smoking machine (SCSM, CH Technologies Inc., Westwood, NJ, USA) following previously published methods [29, 30] (see also Fig. 1). Animals were exposed to TS mixed with oxygenated air six times/day; two cigarettes/h, 6–8 h/day, 7 days/week for 3 weeks according to the International Organization for Standardization/Federal Trade Commission (ISO/FTC) standard smoking protocol. This latter consists of 35 ml puff volume, 2-s puff duration, 58-s intervals, eight puffs per cigarette [31]. Control and TBI animal groups underwent the same operational procedure (to minimize the variability impact of the process itself on behavioral assessments) but were exposed to oxygenated air [30].

### Induction of head injury in mice

We used a weight-drop model of TBI [4, 32]. This model was developed to mimic human closed head injury (CHI) by a standardized weight-drop device inducing a focal blunt trauma over an intact skull without pre-injury manipulations. The impact triggers a robust neuroinflammatory response (highly reproducible), BBB breakdown, and neurological impairment [33].

In brief, mice were first anesthetized by inhaling isoflurane vapor for 3 to 5 min and then placed on a spongy platform positioned under the weight-drop device. Head movements were allowed parallel to the injury plane at the time of the induction to mimic a mild to moderate head injury. During the induction phase, mice were positioned to direct the trauma from the left anterior frontal area at the same distance between the eye and the ear. A hollow tube (with an internal diameter of 13 mm) was used as the guiding system for a metal weight (30 g) released in a free fall from the dropping point positioned 80 cm above the target (Fig. 1). The sham-injured mice underwent the same procedures with the exclusion of being subjected to head impact by weight drop.

### Open field test

Open field test is a standard measure of exploratory behavior and general activity in rodents [34]. Briefly, mice were housed into 16″ × 16″ unobstructed glass chamber containing infrared sensors along the perimeter. Then the mice were monitored and recorded for 1 h, and the first 30 min of 1 h were excluded as the acclimatization period (Fig. 1). Automatic calculation of the activity (total distance traveled) and resting time of the animals was performed by Versamax software (Accuscan Instruments, Columbus, OH). All behavioral tests were performed between 9 am and 1 pm.

### Blood collection and brain isolation

Mice were sacrificed under terminal anesthesia 3 days after TBI to collect blood samples and brains for subsequent biochemical and molecular analysis. Blood samples were collected by cardiac puncture as described elsewhere [35, 36]. Briefly, mice were positioned on their back. All animals were anesthetized with inhaled isoflurane (4% induction; 1–1.5% maintenance) to minimize discomfort, distress, and pain. Then a V-cut was made through the skin and abdominal wall, and internal organs were moved to the side. The needle was inserted through the diaphragm and into the heart. Blood was collected by applying negative pressure on the syringe plunger. To isolate the brain, we cut at the nape and then extended along the midline from the dorsal cervical area to the tip of the nose. The skin was then pulled away from the skull laterally. The skull was cut and opened by placing the point of the scissors in the foramen magnum and cutting along the midline. After levering away of parietal bones from the brain and disrupting the nerve attachments at the brain stem and the optic chiasm, the brain was removed from the skull into the sterile medium [21, 29].

### Preparation of protein extracts and Western blotting

Homogenized brain tissues were lysed using RIPA lysis buffer. The total protein content (including nuclear, cytosolic, and membrane fractions) was collected by centrifugation at 14,000 g for 30 min. Samples were then aliquoted and stored at − 80 °C for subsequent protein expression analysis by Western blotting. Protein quantification was conducted using Pierce BCA Protein Assay Kit (Thermo Scientific, # 23225). Samples (60–90 μg for tissue lysates) were prepared as previously described by us [22, 29, 37]. Briefly, denatured samples were run on SDS-PAGE (4–15% gradient gel) and transferred to polyvinylidene fluoride (PVDF) membranes or nitrocellulose membranes for further blotting. The membranes were washed with Tween-Tris-buffered saline (TTBS) (10 mmol/l Tris-HCl, pH 7.4, 150 mmol/l NaCl containing 0.1% Tween-20), then blocked for 1 h with Tween-TBS (containing 5% non-fat dry milk), and incubated overnight at 4 °C with primary antibodies prepared in TTBS containing 5% bovine serum albumin (BSA). The following day, cells were washed and then incubated with the secondary antibody prepared in Tween-TBS containing 5% BSA for 2 h. The protein band densities were visualized using chemiluminescent reagents according to the manufacturer’s instructions. We used Image Studie Lite Ver 3.1 for protein quantification analysis. All protein quantifications were adjusted for the corresponding β-actin level and reported as fold changes vs. control.

### Enzyme-linked immunoassay

Blood samples collected from mice were analyzed by Quantikine ELISA kits (R & D systems, Minneapolis, MN, USA) for the quantitative determination of thrombomodulin and cytokines TNF-α, IL-6, and IL-10 according to the procedure per the manufacturer’s protocol.

### Measurement of glutathione levels

Tissue lysate was analyzed by Quantification Kit for Oxidized and Reduced Glutathione (Sigma Aldrich, St. Louis, MO, USA) according to the manufacturer’s guidelines. For the quantitative determination, samples were prepared by lysis of total cell protein in T-PER lysis buffer followed by dilution of 1:50 for glutathione (GSH) analysis. In brief, a serial dilution of reduced (GSH) and oxidized glutathione (GSSG) stock standards were prepared along with assay mixtures for detection of GSH and total GSH using 100 X Thiol green stock solutions, assay buffer, and GSSG probe. A one-step fluorometric reaction of the sample with the respective assay buffer was incubated for 30 min. Fluorescence intensity was assessed at Ex/Em of 490/520 nm. GSSG was determined by subtracting GSH from total GSH. Finally, GSH was plotted against GSSG to obtain the GSH/GSSG ratio.

### Statistical analysis

All collected data were expressed as mean, standard ± deviation (SD). The sample size was chosen based on previous work by us and others to generate 80% power and a type 1 error rate = 0.05. The blind analysis was performed by one-way ANOVA using GraphPad Prism 8 Software Inc. (La Jolla, CA, USA). Post multiple comparison tests were performed as with Tukey’s or Dunnett’s test as recommended by the software. *P* values < 0.05 were considered statistically significant.

## Results

As shown in Fig. 1, TS generated by a CSM-SCSM cigarette smoking machine (CH Technologies, Westwood, NJ) was forced directly into two airtight smoking chambers measuring 24 L × 12 W × 12 H. The smoking inlet is dually connected to a feeding tube and a ventilator system supplying O_2_ (2 L/min) at atmospheric pressure (1 bar). Mice were housed in the smoking chambers (four mice/chamber), receiving an uninterrupted supply of normal oxygenated air in between puffs. At the end of each smoking cycle, mice were transferred immediately back to their regular housings with standard food and water supply.

### TBI and TS exposure negatively affect body weight

**Fig. 2:**
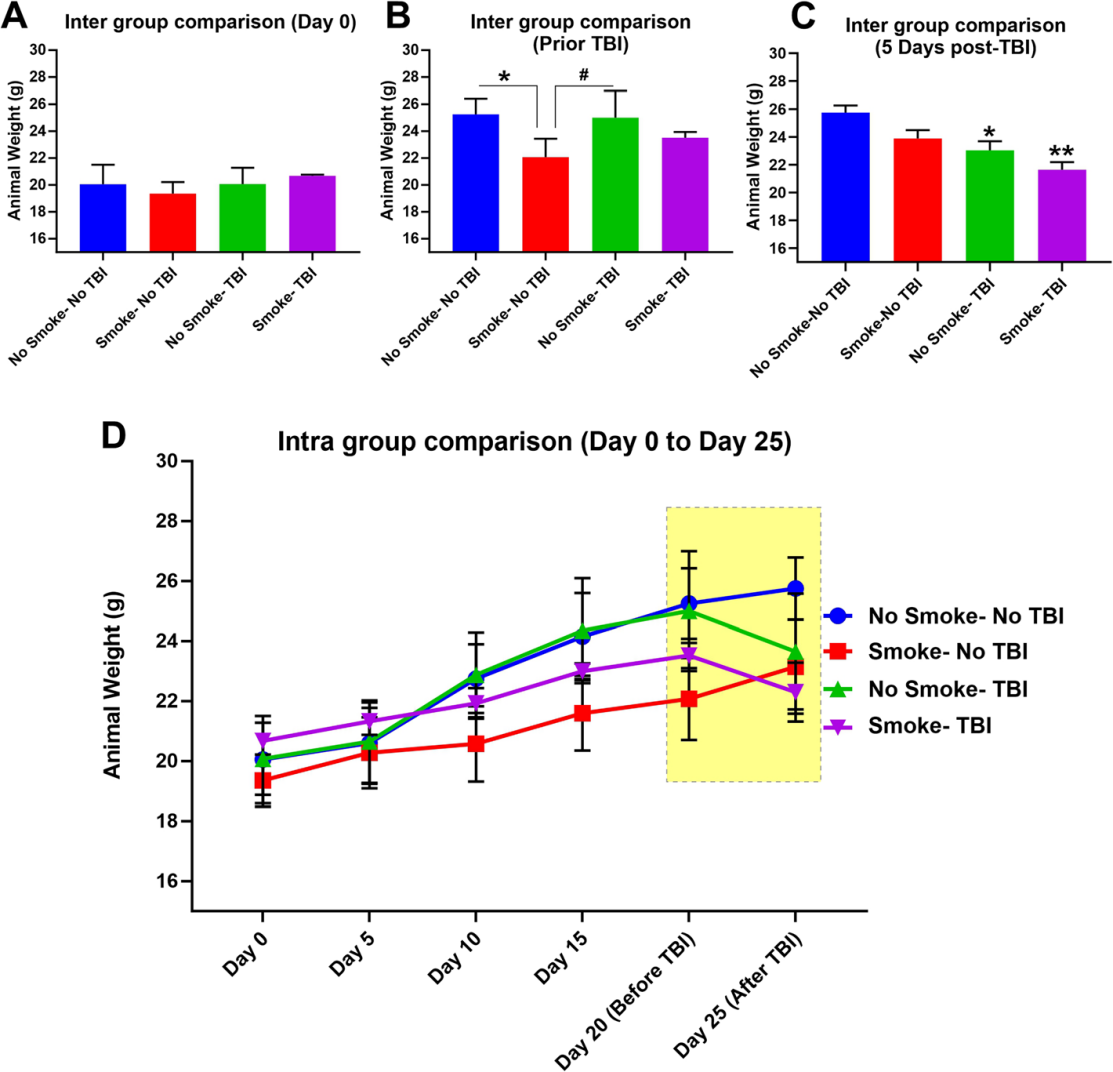
Effect of premorbid TS-exposure and TBI on body weight in vivo. **a** Measurements of animals’ body weight do not show any significant difference between the tested groups at day 0. However, at the end of the 3 weeks of exposure before TBI, **b** animals exposed to TS showed a decreased body weight when compared to controls. **c** Three days after TBI, animals showed significantly decreased body weight compared to controls. **d** Longitudinal assessment of animals’ body weight (all groups). *n* = 6 biological replicates for each experimental group. *p < 0.05 vs. control. #*p* < 0.05 0001 vs. smoke

Weight analysis was regularly performed to assess whether smoke and/or TBI had any negative impact on body weight. Animals were divided into four groups at day 0 (Fig. 2a) representing the four main test categories: control (no smoke exposure and no TBI), smoke (but no TBI), TBI (without smoke exposure), and smoke exposure followed by TBI (smoke + TBI). Each group consisted of six animals. As shown in Fig. 2b, we observed that chronic TS exposure alone was enough to reduce the growth rate of body weight over time. This data is consistent with published data likely conducible to an appetite suppressant effect as well as a moderate increase of metabolism promoted by TS [29]. Following TBI, we also observed a post-traumatic reduction in body weight in animals that underwent TBI with and without TS exposure (Fig. 2c). Note also that post-TBI animals did not receive any further TS exposure. The full longitudinal study data pattern has been reported in Fig. 2d.

### TS exposure and TBI promote vascular inflammatory responses and potentially impact blood hemostasis

**Fig. 3:**
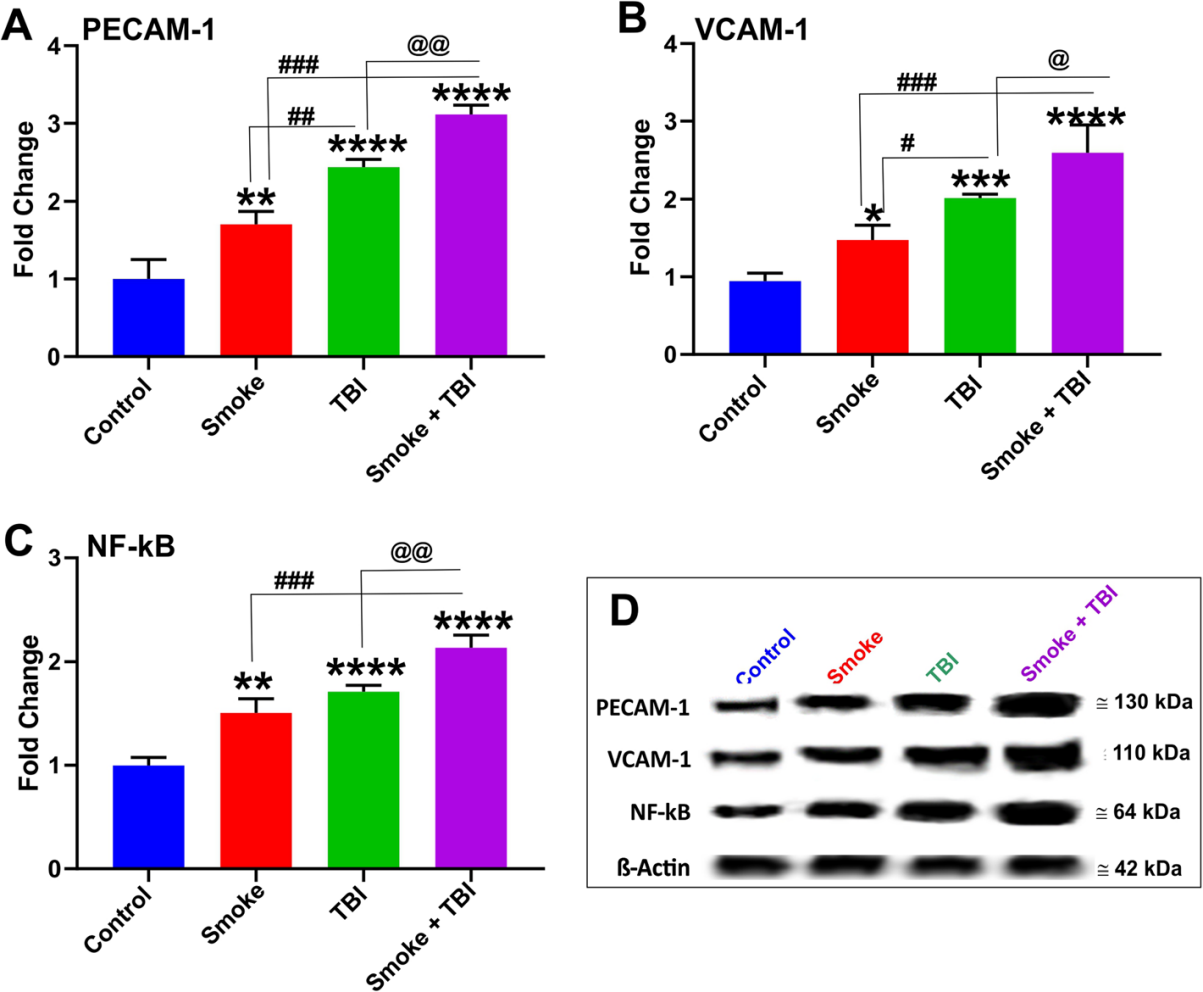
Effect of premorbid TS exposure and TBI on vascular inflammatory responses. **a** Expression level of the inflammatory adhesion molecules PECAM-1 and **b** VCAM-1 and the pro-inflammatory regulator NF-kB **c** which were upregulated by TS-exposure and synergistically potentiated by TBI. *n* = 6 biological replicates for each experimental group. *p < 0.05, **p < 0.01, ***p < 0.001, ****p < 0.0001 vs. control. #*p* < 0.05, ##*p* < 0.01, ###*p* < 0.001 vs. smoke. @*p* < 0.05, @@*p* < 0.01 vs. TBI. WB analyses report protein/β-actin ratios

**Fig. 4:**
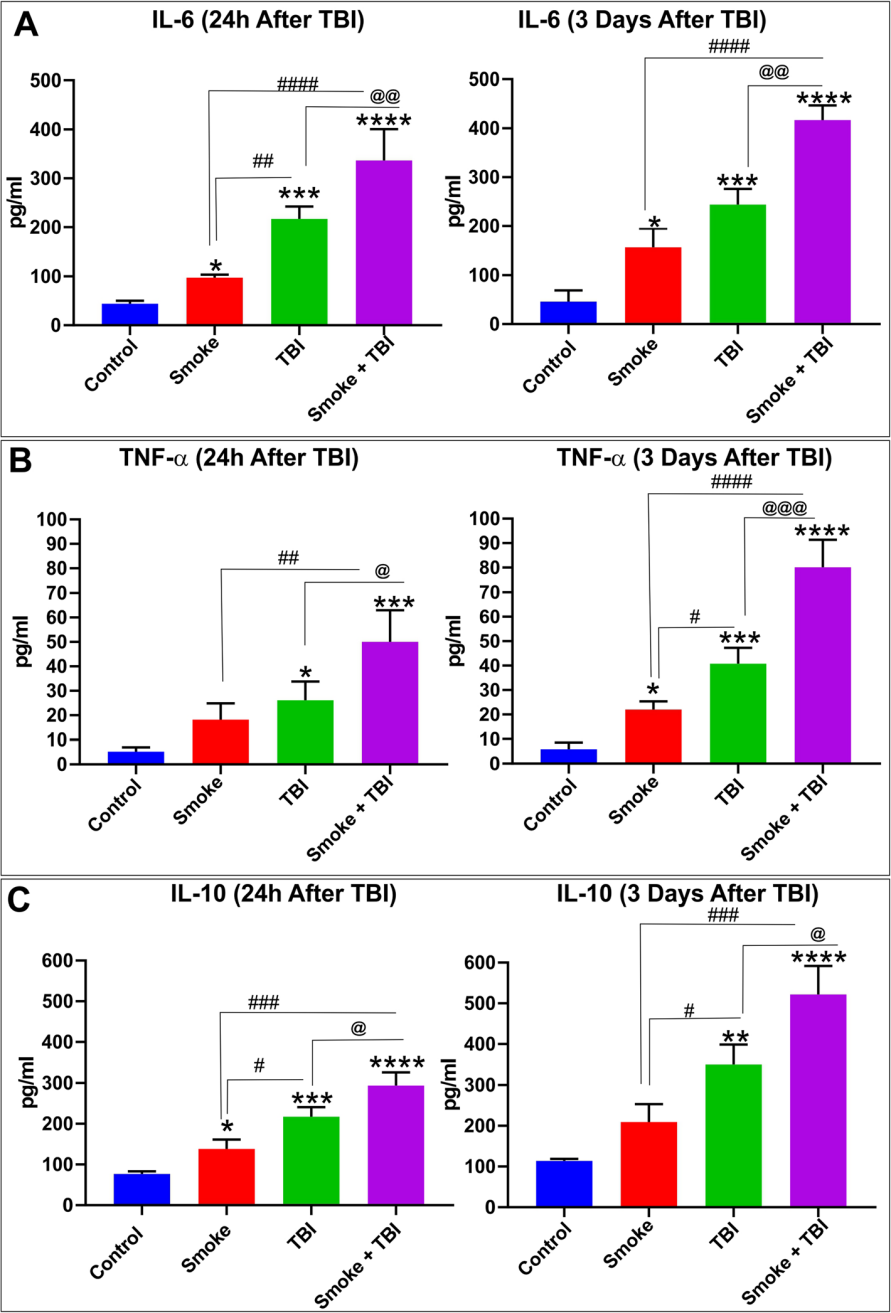
Effect of premorbid TS exposure and TBI on pro-inflammatory cytokines IL-6, IL-10, and TNF-α. ELISA results of pro-inflammatory cytokines **a** IL-6, **b** TNF-α, and **c** IL-10 24 h and 3 days after TBI. Our data shows TS exposure promoting the inflammation and synergistically potentiating the cytokines upregulation induced by TBI. *n* = 6 biological replicates for each experimental group. *p < 0.05, **p < 0.01, ***p < 0.001, ****p < 0.0001 vs. control. #*p* < 0.05, ##*p* < 0.01, ###*p* < 0.001, ####*p* < 0.0001 vs. smoke. @*p* < 0.05, @@*p* < 0.01, @@@*p* < 0.001, @@@@*p* < 0.0001 vs. TBI

Our primary hypothesis refers to the alteration in pro-inflammatory markers as one of the mechanisms that underlie the damage in the brains of TBI-induced mice. Hence, we evaluated the expression levels of inflammatory marker NF-kB, inflammatory adhesion molecules VCAM-1, and PECAM using Western blotting and enzyme-linked immunoassay (ELISA). As shown in Fig. 3, Western blotting analysis revealed a significant increase in the expression level of PECAM-1 (Fig. 3a) as well as vascular adhesion molecules VCAM-1 (Fig. 3b), and NF-kB (Fig. 3c) in mice exposed to TS with or without TBI as well as mice undergoing TBI alone. As evident from the data analysis and the exemplative blots (Fig. 3d), both TS and TBI elicited an inflammatory response as standalone stimuli. However, the effect was significantly increased, whereas the two stimuli were combined. Our results suggest a synergistic effect between TS and TBI. Furthermore, as shown in Fig. 4, the inflammatory activity of TBI and TS were confirmed by the analysis (via ELISA) of the pro-inflammatory cytokines IL-6 (Fig. 4a), IL-10 (Fig. 4b), and TNF-α (Fig. 4c) in blood samples collected 24 h and 72 h after TBI. Similar to NF-kB and the adhesion molecules, we observed a synergistic effect between TS and TBI.

**Fig. 5:**
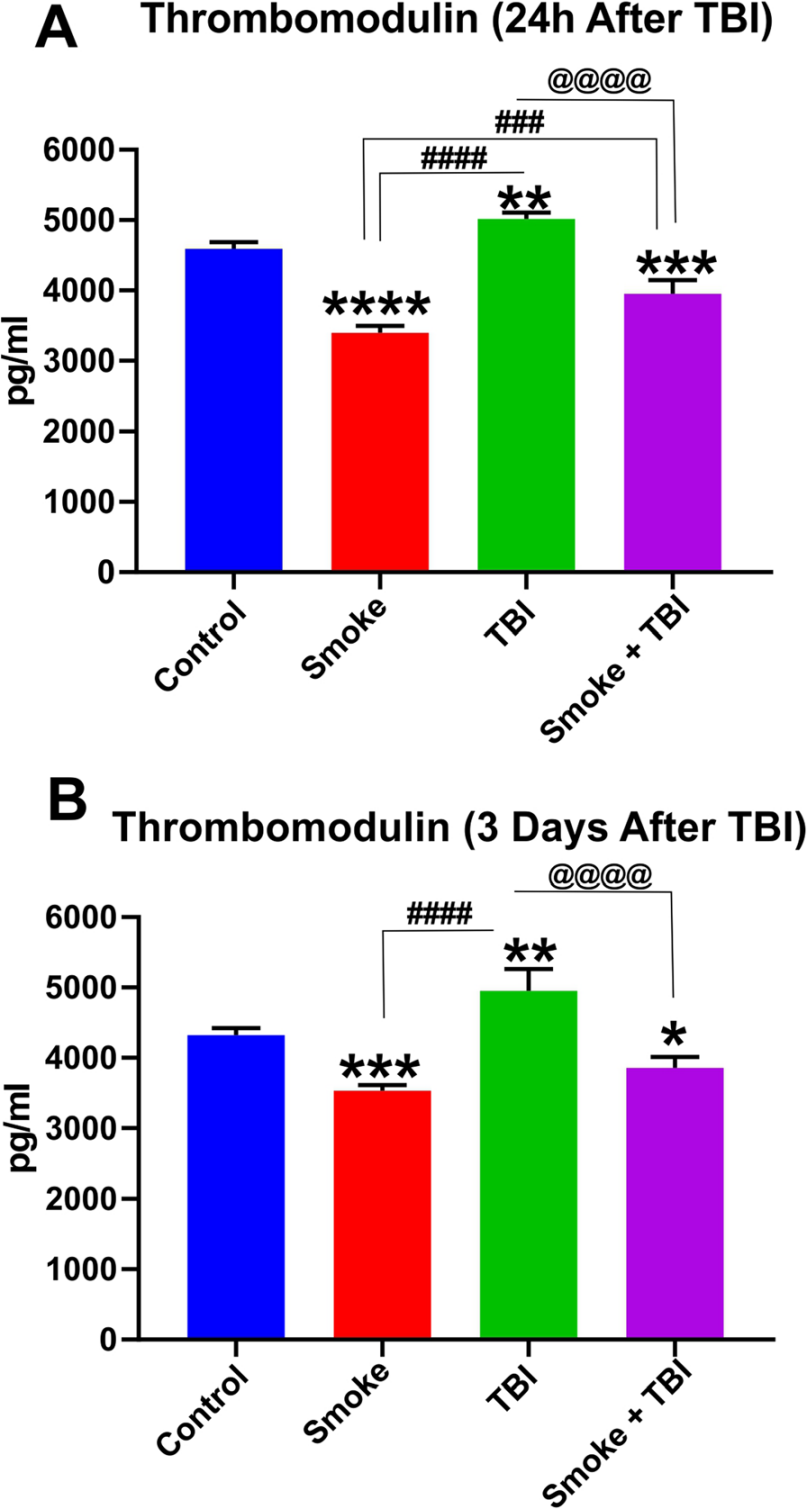
Effect of premorbid TS exposure and TBI on plasma level of thrombomodulin. ELISA measurement of thrombomodulin levels in the blood samples collected at **a** 24 h after TBI and **b** 3 days after TBI. Note how TS exposure promotes downregulation of thrombomodulin and therefore potentially impairing blood hemostasis such as the control of the blood coagulation cascade. *n* = 6 biological replicates for each experimental group. *p < 0.05, **p < 0.01, ***p < 0.001, ****p < 0.0001 vs. control. ###*p* < 0.001, ####*p* < 0.0001 vs. smoke. @@@@*p* < 0.0001 vs. TBI

Of relevant interest, TS exposure decreased the expression level of the anticoagulant factor thrombomodulin (Fig. 5a, b). This effect has been previously observed both in vitro and in vivo [29, 30]. By contrast, thrombomodulin was upregulated by TBI as a standalone factor; however, it remained downregulated in TBI animals pre-exposed to TS. The effect was statistically significant, even 72 h (3 days) post-TBI (Fig. 5b).

### Downregulation of NRF2 and its downstream effector NQO-1 and HO-1 by TS exposure and TBI

**Fig. 6:**
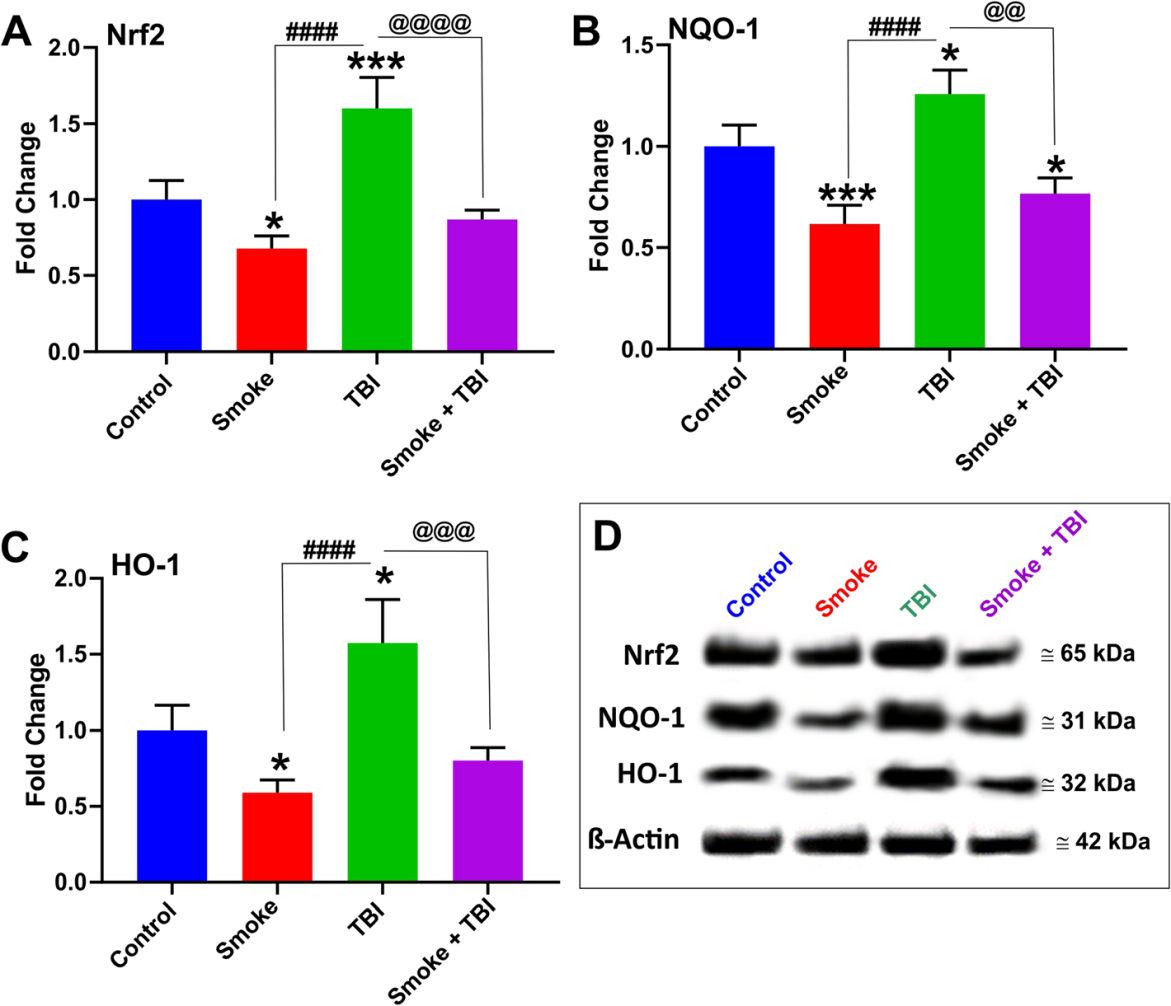
Effect of premorbid TS exposure and TBI on the antioxidative response system. **a** Western blotting analysis emphasizing the effect of chronic TS exposure on Nrf2 expression level as a standalone factor and in synergism with TBI. **b**, **c** Changes in Nrf2 expression levels were paralleled by corresponding changes of its downstream detoxifying effector molecules NQO-1 and HO-1. **d** Representative blot images of the analytes. *n* = 6 biological replicates for each experimental group. *p < 0.05, ***p < 0.001 vs. control. ####*p* < 0.0001 vs. smoke. @@*p* < 0.01, @@@*p* < 0.001, @@@@*p* < 0.0001 vs. TBI

The effect of TS exposure and TBI on the expression of the antioxidative response nuclear factor erythroid-2-related factor 2 (Nrf2) was also evaluated by Western blot analysis of whole-brain homogenate, as shown in Fig. 6. Consistent with our previous finding, chronic TS exposure significantly downregulated Nrf2 expression (Fig. 6a) and that of its immediate downstream effector including NAD(P)H dehydrogenase [quinone] 1 (NQO-1; Fig. 6b) and Heme oxygenase 1 (HO-1; Fig. 6c), thus suggesting impairment of the antioxidative response system. By contrast, TBI, as a standalone stimulus, had the opposite effect were Nrf2 was instead upregulated. These data are also evident from the Western blotting shown in Fig. 6d. Although cellular localization (cytoplasmic vs. nuclear distribution) was not possible at this time, the fact that its immediate effectors were also similarly upregulated suggests that the activity of the Nrf2 antioxidative response system was indeed increased following TBI but abrogated by TS exposure. This latter further confirms the detrimental impact of chronic smoking in post-traumatic brain injury settings.

### Synergistic impact of combined chronic TS exposure and TBI on oxidative stress

**Fig. 7:**
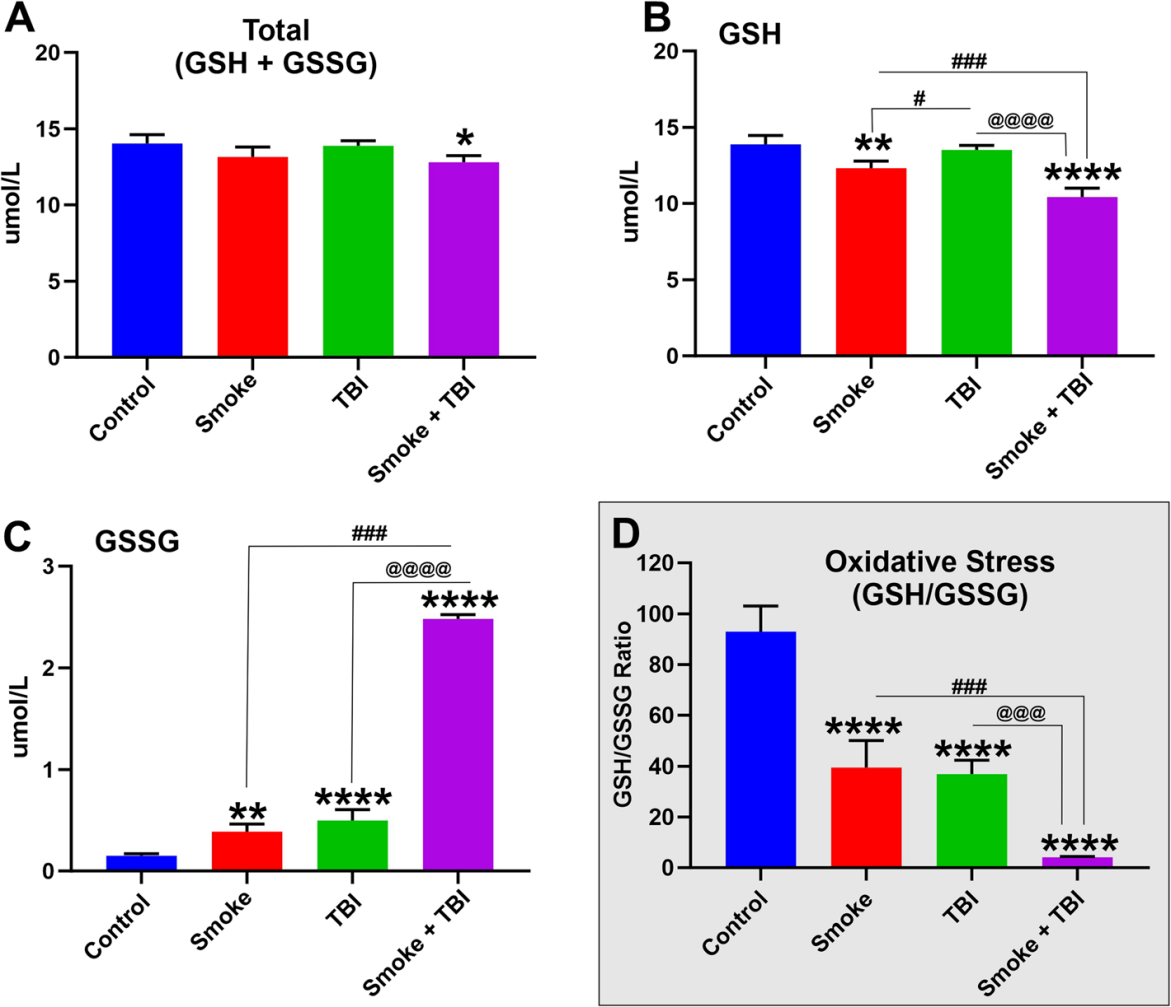
Effect of premorbid TS-exposure and TBI on glutathione levels in vivo. **a** Total glutathione (GSH + GSSG), **b** reduced glutathione (GSH), **c** oxidized glutathione (GSSG), and **d** GSH/GSSG measured by plate reader. *n* = 6 biological replicates for each experimental group. *p < 0.05, **p < 0.01, ***p < 0.001, ****p < 0.0001 vs. control. #*p* < 0.05, ##*p* < 0.01, ###*p* < 0.001, ####*p* < 0.0001 vs. smoke. @*p* < 0.05, @@*p* < 0.01, @@@*p* < 0.001, @@@@*p* < 0.0001 vs. TBI

Our results show that except for the smoke + TBI condition, the overall total glutathione levels are not significantly different from controls (Fig. 7a). Premorbid TS exposure as a standalone factor, as well as the combination of TS premorbid condition with TBI, promote a significant decrease in GSH levels (see Fig. 7b). In line with these results, GSSG levels (oxidated glutathione indicative of elevated oxidative stress responses) are significantly elevated in all the tested conditions when compared to controls (Fig. 7c). In this setting, premorbid TS exposure coupled with TBI produces a substantially higher level of GSSG than either smoke or TBI (Fig. 7c). The impact of TS, TBI, and the combination of both on glutathione levels is also evident from the calculated GSH/GSSG ratio shown in Fig. 7d.

### Chronic TS exposure hamper BBB integrity in TBI

Additional experiments were performed to assess the effect of TS exposure and TBI on BBB integrity. We evaluated the expression level of the BBB accessory protein zonula occludens-1 (ZO-1) and tight junction (TJ) protein expression encompassing the primary regulator of BBB paracellular permeability Occludin and Claudin-5.

**Fig. 8:**
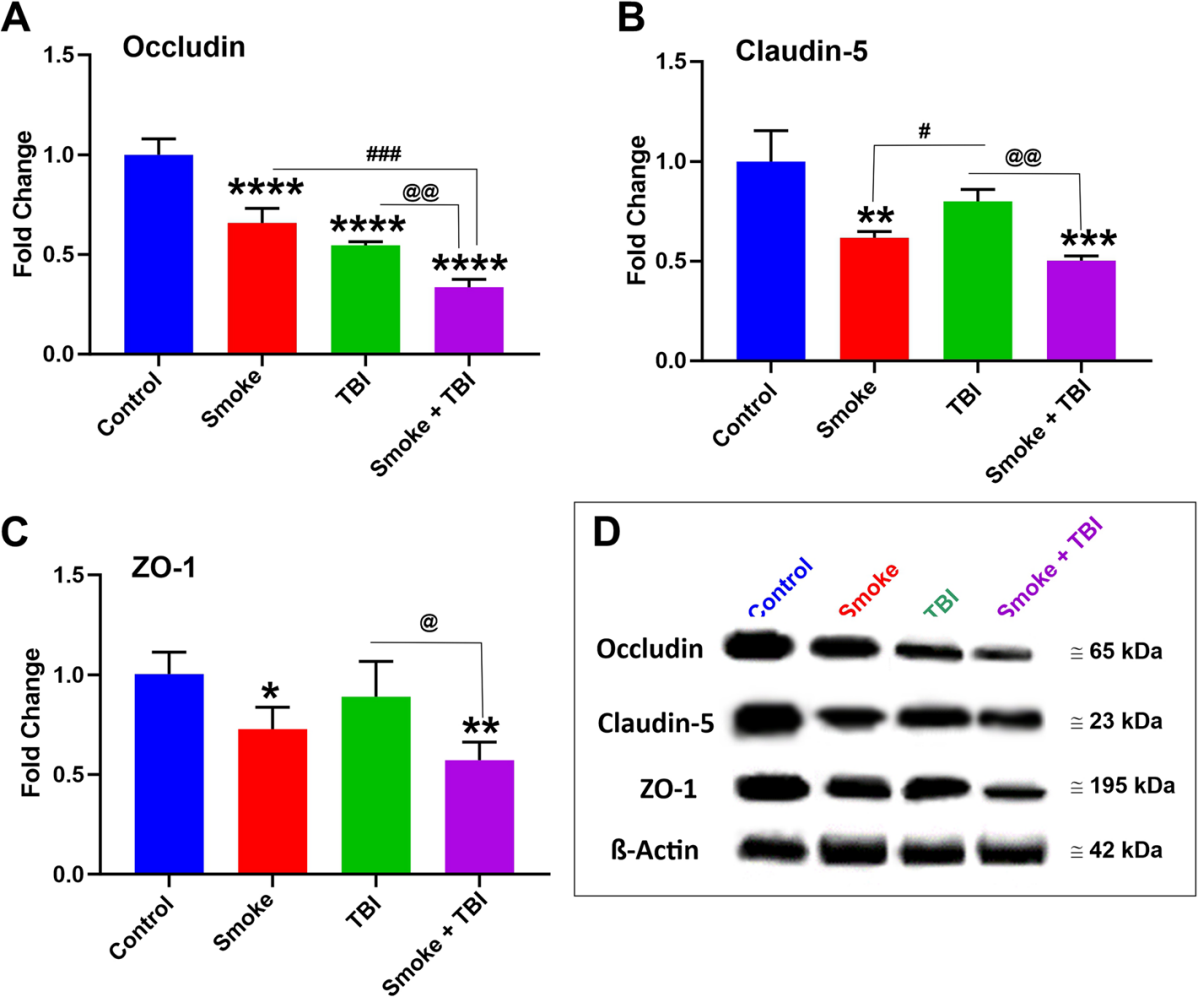
Effect of premorbid TS-exposure and TBI on BBB integrity. A Western blotting analysis demonstrating downregulation of TJ proteins Occludin (**a**), Claudin-5 (**b**), and accessory anchoring protein ZO-1 (**c**) in mice exposed to TS and/or TBI. Note that TBI per se had only a marginal impact on the expression levels of claudin-5 and ZO-1. Therefore, with the exclusion of occludin, which was significantly downregulated by TBI, TS seemed mainly responsible for the TJ downregulation observed in TBI mice chronically exposed to cigarette smoke. *n* = 6 biological replicates for each experimental group. *p < 0.05, **p < 0.01, ***p < 0.001, ****p < 0.0001 vs. control. #*p* < 0.05, ####*p* < 0.0001 vs. smoke. @*p* < 0.05, @@*p* < 0.01, vs. TBI

As shown in Fig. 8, chronic TS exposure significantly downregulated the expression of Occludin (Fig. 8a), Claudin-5 (Fig. 8b), and ZO-1 (Fig. 8c) when compared to controls (see also Western blots grouped in Fig. 8d). These results are consistent with previous work by our group, suggesting that TS acts as the main effector of TJ downregulation, thus promoting the loss of BBB integrity [29, 31, 37].

By contrast, TBI only has a significant impact on occludin (which is a crucial regulator of BBB integrity). At the same time, the effect on claudin-5 was marginal, and no effect was observed regarding the expression of ZO-1. However, when the stimuli are combined, we see a synergistic downregulation of occludin, whereas that of claudin-5 and ZO-1 is attributable uniquely to TS exposure. The critical point of these results is that in addition to inflammation, premorbid TS exposure promotes the onset of a pre-existing state of weakened BBB integrity, which can be more readily impacted by TBI.

### TS promotes increased motor activity in mice but aggravates post-traumatic behavior in mice undergoing TBI

Exploratory behavior and general activity of the test mice were regularly recorded to evaluate the impact of smoke and TBI based on their motor activity before and after induction of a mild traumatic injury.

**Fig. 9:**
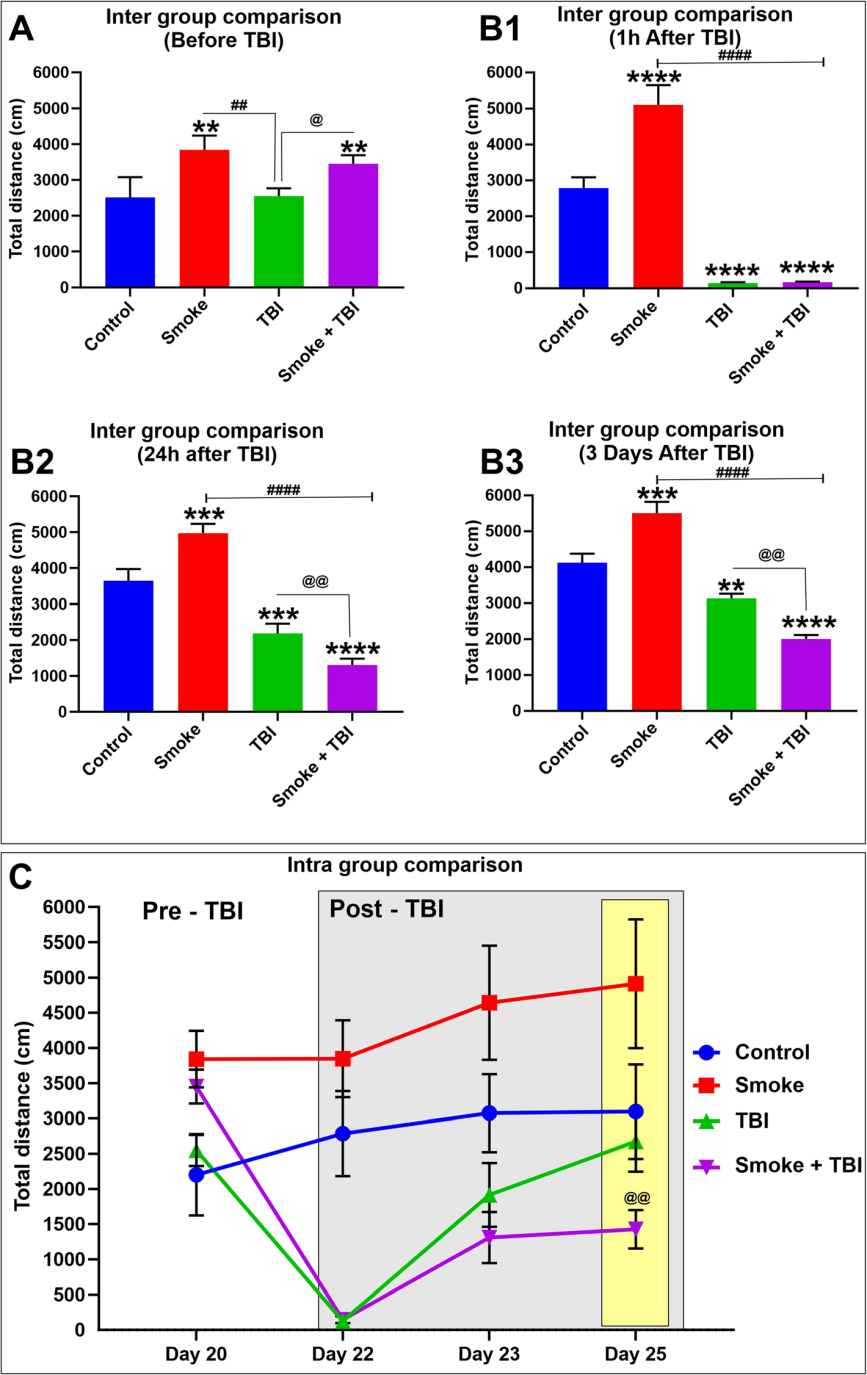
Effect of premorbid TS-exposure and TBI on exploratory behavior and general motor activity. (A) Measurements of totaled distance traveled by mice do not show any significant difference between the tested groups at day 0 before TBI. However, mice undergoing chronic TS exposure w/o TBI demonstrated a significantly higher motor activity (B1). Note also that both TBI and TS-exposed mice undergoing TBI displayed a significant reduction in motor activity. Measurements performed at 24 and 72 h post-TBI clearly show that TS further aggravated TBI injury as denoted by the significant reduction of motor activity when compared to the TBI mice group (B2 and B3). (C) Longitudinal assessment of the animals’ response to injury w/wo TS exposure clearly shows that animals chronically exposed to TS experienced a significant delay in motor activity recovery when compared to TBI animals that were not exposed to TS. *n* = 6 biological replicates for each experimental group. *p < 0.05, ***p < 0.001, ****p < 0.0001 vs. control. ##*p* < 0.0, ####*p* < 0.0001 vs. smoke. @*p* < 0.05, @@*p* < 0.01 vs. TBI

As shown in Fig. 9, we observed that by comparison to both groups of TS-free mice, animals chronically exposed to TS demonstrated substantially higher motor activity expressed as total distance traveled by the animals. This data is consistent with the metabolic effect of TS as well as a craving for TS itself (Fig. 9(B1 to 9B3)). We expected that TBI would reduce the mice’s motor activity (the effect was evident as early as 1 h post-trauma and partially recovered later on). Interestingly, behavior/activity was even more significantly reduced in TBI mice chronically exposed to TS when compared to TBI alone (see Fig. 9(panels B2 and B3)). Furthermore, as shown in Fig. 9 panel C (yellow insert), TS exposure also negatively impacted motor recovery when compared to TBI mice that were not exposed to TS and which returned to levels comparable to controls within three days post-TBI.

**Fig. 10:**
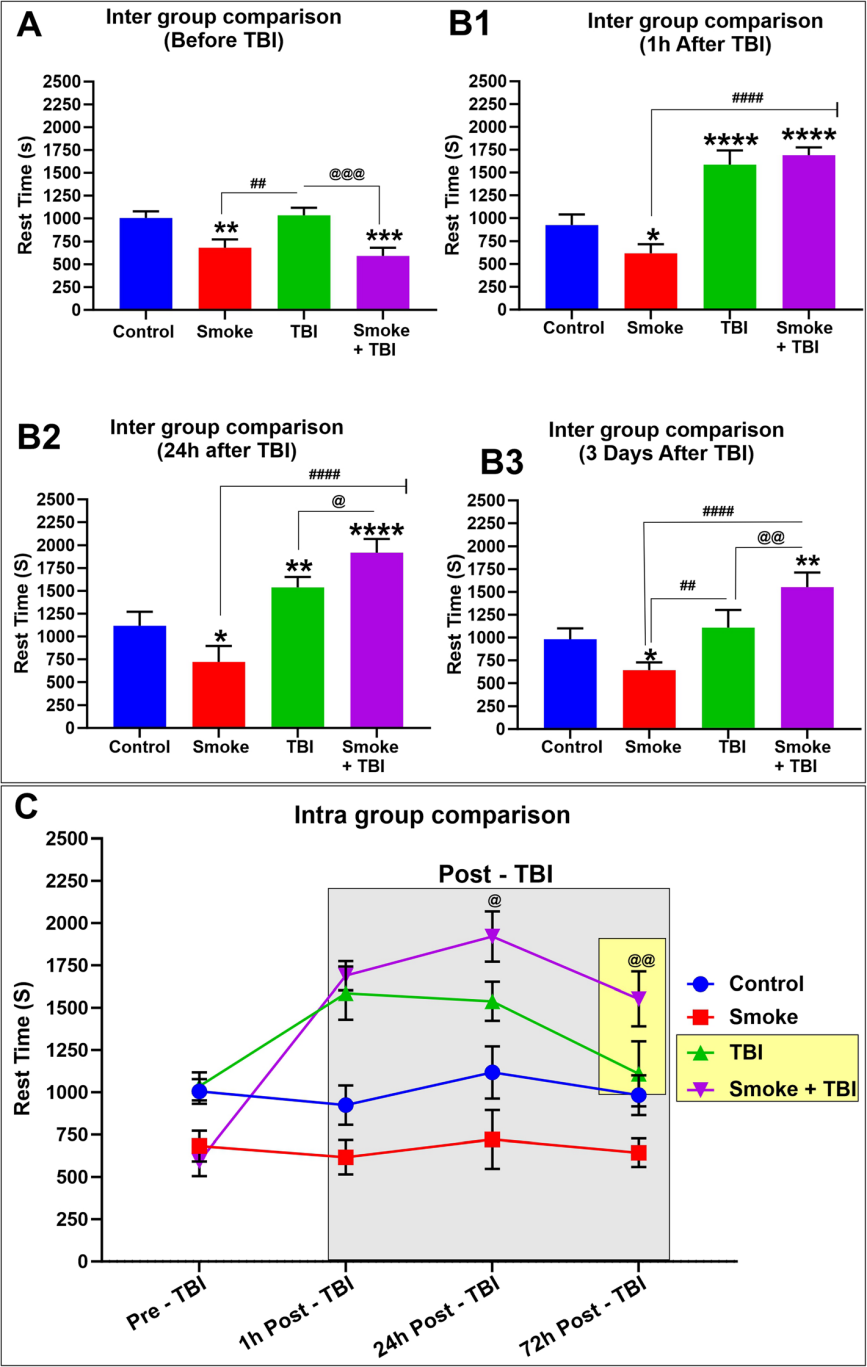
Additional effect of premorbid TS-exposure and TBI on general motor activity such as resting time. (A) Intergroup rest time comparison prior TBI shows the effect of chronic TS exposure on motor activity. In this specific case, the increased rest time observed in smoke and smoke + TBI animal groups well match the increased motor activity (distance traveled) previously assessed. (Panel B1 to B3) TS increases rest time of animals undergoing TBI at 24- and 72-h post injury when compared vs TBI alone. (Panel D) Side by side longitudinal assessment of rest time between the 4 test groups. *n* = 6 biological replicates for each experimental group. *p < 0.05, **p < 0.01, ***p < 0.001, ****p < 0.0001 vs. control. ##*p* < 0.0, ####*p* < 0.0001 vs. smoke. @*p* < 0.05, @@*p* < 0.01, @@@*p* < 0.001 vs. TBI

Moreover, as shown in Fig. 10 (panels A through C), the rest time follows the opposite pathway of motor activity, where mice chronically exposed to TS exhibited a significantly reduced rest time compared to controls. Note also how the rest time significantly increases following TBI and renormalizes after 72 h (see Fig. 10 (panel D)). Of significance is the fact that mice undergoing chronic premorbid TS exposure before TBI exhibited a significantly higher rest time than TBI at both 24 and 72 h post-injury. These results match very well the motor activity assessments previously discussed. The increased rest time in TS-exposed mice undergoing TBI vs. non-treated TBI animals reflects the pathogenic impact afforded by TS in addition to that induced by TBI.

## Discussion

TBI is a global health issue and one of the major leading causes of death and disability for individuals under the age of 50. According to the statistics, approximately half the global population will experience one or more TBI events over their lifetime [8]. Oxidative stress caused by the redox imbalance promoted by highly reactive oxygen species (ROS, including free oxygen radicals and reactive anions) has been proven to play a significant role in post-traumatic secondary brain damage [19, 21]. Recent studies have shown that TS exposure is associated with impairment of the antioxidative response system and dysfunction of normal endothelial physiology. Therefore, TS acts as a prodromal factor for the onset of major cerebrovascular, neuroinflammatory, and degenerative disorders [24, 30, 31, 38–41]. However, there is still considerable controversy regarding the influence of cigarette smoking as a commonly premorbid factor on how it relates to TBI and its impact on post-traumatic secondary brain injury and post-TBI recovery [3].

In the present study, we evaluated the potential influence of chronic tobacco smoking on pathophysiological mechanisms underlying the exacerbation of TBI and retardation of post TBI recovery using a weight-drop mice model. In this model, a fixed weight is released for a free fall based on a defined path and height. The weight and the height from which the weight is dropped determine the severity of the injury, which can range from mild to severe. This model was chosen because of its ability to simulate traumatic head injuries comparable to those observed in road accidents or falls [4]. Based on our results, both groups of smoked mice demonstrated a loss of body weight when compared to control, confirming the common metabolic stimulatory effect of TS. Longitudinal increase of body weight was also significantly dampened after TBI induction, which is consistent with the well-observed reduced appetite after TBI. In line with these findings, the behavioral analysis confirmed similar changes in the state of consciousness and awareness immediately after TBI and during recovery. While TS was consistently associated with an increase in motor activity as a standalone stimulus, when it was combined with TBI (see Fig. 9), it further depressed motor activity when data were compared to TBI mice that were not exposed to TS. The post-traumatic motor recovery was also significantly reduced when compared against the same group. These data are consistent with the increased severity of post-traumatic brain injury promoted by TS and well correlated with the analysis of inflammatory biomarkers as well as BBB integrity.

Moreover, systemic and microenvironmental effects of neuroinflammation induced by TBI can affect neurotransmission and especially the dopaminergic pathways. This latter plays a significant role in secondary injury as well as behavioral and cognitive dysfunction following TBI [42–44]. Previous studies suggested that dopamine dysregulation may have a substantial role in behavioral deficit after TBI, which might be due to alterations in proteins regulating the dopamine [45, 46]. Dopamine release is suppressed after TBI, and a higher dosage of the substance has been necessary to maintain the behavior. Moreover, alterations in dopaminergic pathways have also been evidenced by increases in tobacco abuse after TBI since nicotine is the principal component of tobacco smoke that underlies the addictive properties of cigarette smoking [47, 48]. In chronic smokers, nicotine increases the release of dopamine by stimulating nicotinic cholinergic receptors (nAChR) in multiple regions of the brain. Cravings and aberrant behaviors may be initiated and promoted because of the enhanced dopamine release after a long-standing TS experience. On the other hand, higher nicotine exposure may cause rapid nAChR desensitization, which induces nAChR function loss [49]. Thus, chronic tobacco smoking leads to the development of addiction, which is linked to dysfunctions in the DA transmission system [49].

Nrf2, a basic region-leucine zipper (bZip) redox-sensitive transcription factor, is the master regulator of multiple cytoprotective responses which controls the redox state of cells in harmful stresses [21, 50, 51]. Under normal conditions, Nrf2 is localized in the cytoplasm by its inhibitor, Kelch-like ECH-associated protein 1 (Keap1). Nevertheless, under conditions of oxidative or xenobiotic stress, the cysteine residues of Keap1 become oxidized, and Nrf2 dissociates from Keap1. The free Nrf2 then translocates into the nucleus, binding to the antioxidant response element (ARE), thus promoting gene transcription. This latter includes over 500 genes encompassing phase 1 and 2 enzymes, regulators of redox metabolism, production of ATP and antioxidative agents (including NADH and glutathione), and TJ expression at the BBB [16, 52, 53]. Based on valid evidence, Nrf2 also promotes anti-inflammatory mediators, the activity of the proteasome, and other transcription factors involved in mitochondrial biogenesis [54]. According to recent studies, suppression of Nrf2 activity and impairments of the Nrf2–ARE pathway exacerbate TBI-induced oxidative damage as well as post-traumatic neurological deficits. This data strongly suggests that Nrf2 plays a significant neuroprotective role in TBI and neurodegenerative disorders [4, 11]. Since the upregulation of Nrf2 activity reduces the burden of TBI-induced brain injury, it is plausible that positive modulation of Nrf2 could better TBI outcomes through the reduction of oxidative stress, inflammation, and protection of BBB integrity [29, 30, 55–59].

In line with these findings, we assessed the impact of premorbid TS exposure and TBI on Nrf2 expression levels, as well as its downstream effector molecules NQO-1 and HO-1, which are known for exerting acute detoxification and cytoprotective functions. Our in vivo data show that the Nrf2-ARE system is activated in response to TBI (see Fig. 6). This effect could be due to a direct modulatory activity toward Nrf2 expression and the sequential activation of the Nrf2–ARE pathway in response to trauma. Our findings are in line with the results obtained in a previous study by Li et al., indicating significantly enhanced Nrf2, NQO-1, and HO-1 protein expression following TBI [58]. However, chronic TS exposure as a standalone stimulus has the opposite effect (see Fig. 6a), which is also in agreement with previous in vitro and in vivo observations recently published by our group [26, 29, 30, 60].

The oxidative stress impact of premorbid chronic smoking and TBI (as standalone or combined factors) was also confirmed by measurements of GSH as well as GSSG (see Fig. 7). Our data show that TS exposure as a standalone factor is responsible for a statistically significant decrease in the levels of GSH (see Fig. 7b). The combined effect of premorbid TS exposure with TBI also causes a substantial drop of GSH combined with a significant increase in its oxidated form GSSG (see Fig. 7b–d). These data well reflect the impact of TS on the Nrf2 system previously analyzed and further supporting the notion of a synergistic effect. Noteworthy is the concurrent effect of TS and TBI, which negatively impact the total level of glutathione (GSH + GSSG; see Fig. 7a). This latter suggests that there is a higher turnover of GSH, and its overall production is decreased. GSH production is dependent upon the activity of the Nrf2 system, which promotes glutathione synthesis, among other antioxidative effects [53]. The modest yet significant increase of GSSG observed in TBI (see Fig. 7c) and also evident from the calculated GSH/GSSG ratio (see Fig. 7d) is also indicative of oxidative stress, which in this case is solely mediated by inflammation as shown in Fig. 4. The fact that the level of GSH in TBI does not seem to be diminished when compared to controls (see Fig. 7b) is also in agreement with our results showing upregulation of Nrf2 in response to injury, which promotes GSH synthesis in response to an acute stressor. As a comorbid stimulus, when TS exposure is combined with TBI, it abrogates the post-traumatic activation/upregulation of Nrf2 that follows brain trauma, thus preventing this physiological recovery system from being activated.

The downstream impact of Nrf2 downregulation includes further impairment of the BBB (which was significantly less severe in TBI animals not exposed to TS; see also Fig. 8) and upregulation of post-traumatic inflammatory responses. This latter (including overexpression of the pro-inflammatory transcription factor NF-kB, cytokines, and vascular adhesion molecules; see Figs. 4 and 5) further aggravate vascular integrity and worsen TBI outcome. These findings are consistent with the analysis of post-traumatic motor activity, showing that animals chronically exposed to TS before TBI fared significantly worse than those undergoing traumatic injury but were not exposed to TS (see Figs. 9 and 10). In this specific case, the overall cerebrovascular/BBB impairment promoted by TS before TBI could explain the phenomenon.

The BBB is a complex dynamic interface between the blood and the central nervous system (CNS), which strictly maintains the brain homeostasis and controls the passage of substances in and out of the brain environment. Among the various control functions of the BBB, the inter-endothelial TJs rigidly control the paracellular pathways blocking the passage of polar molecules (including ions) from moving between adjacent endothelial cells [61]. The most critical TJ proteins modulating the extremely low BBB permeability to polar molecules are Occludin and Claudins (more specifically claudin-5), forming homotypic bonding with their corresponding counterparts on adjacent endothelial cells. ZO-1 plays the critical function of anchoring this TJ protein to the cell cytoskeleton, thus allowing the cell to direct the distribution of these TJ proteins around the membrane [62]. Recent findings have demonstrated that BBB impairment is a crucial component of post-TBI secondary brain injury and can significantly affect the outcome [63].

Additional pieces of evidence also indicate that inflammation is an essential contributor to the TBI pathophysiology exacerbating neuronal damage during post-traumatic brain injury. Therefore, sustained and excessive inflammation through the secretion of proinflammatory mediators can increase subsequent neurological impairment [64–66]. This process involves resident microglia and astrocytes, peripheral leukocytes penetrating through the leaking BBB, and inflammatory mediators, including cytokines that interfere with normal restorative processes of the brain, thus promoting neuronal cell death [19, 43, 67]. Proinflammatory cytokines like IL-6, IL-10, and TNF-α are increased in post-traumatic blood samples. Furthermore, the synthesis of chemokines, prostaglandins, and expression of cell adhesion molecules like VCAM-1 and PECAM-1 on the surface of the cerebrovascular endothelium is also increased. This latter process can favor the extravasation of inflammatory cells from the blood into the brain [19, 64]. There is also a growing consensus that all these processes are the principal promoters of the secondary brain damage associated with TBI, including dysfunction of astrocytes and microglia, as well as BBB impairment contributing to the increased paracellular permeability and the loss of neurons [64, 68, 69]. Proinflammatory molecules play a supplementary role in increased BBB permeability related to loss of Occludin/ZO-1 as well as other tight junction [17, 70]. It is also well described that BBB integrity is deeply affected by oxidative stress, so that enhanced ROS production leads to redistribution and/or altered expression of tight-junction proteins, endothelium dysfunction, and increased BBB permeability [71–73]. Inflammation is also linked to oxidative stress, whereas ROS (such as those released within TS) are considered among the most potent inflammatory mediators [74]. Oxidative stress caused by TS has been widely recognized as a negative contributing factor for neurological outcomes following brain injury [55], where TS modulates a cascade of events leading to the activation of NF-κB and the expression of pro-inflammatory cytokines and vascular adhesion molecules [60]. This has been observed in glial cells and neurons following TBI and is associated with long-term inflammatory processes [10]. Mettang et al., using an experimental model of closed-head injury promoted neuronal cell death, demonstrated the repression of the NF-κB inhibitor system exacerbating the neurological outcome and increasing post-traumatic mortality rate [75]. In the contest of Nrf2–NF-κB interplay, recent studies confirmed the cytoprotective activity of Nrf2, which promotes the downregulation of pro-apoptotic mediators such as Bax, BAD, and others. These pro-apoptotic factors are instead upregulated by NF-κB [76, 77]. Nrf2 reduces ROS levels and affects the redox-sensitive NF-κB signaling pathway involved in neuroinflammation.

Moreover, in a recent study, it has been reported that Nrf2^−/−^ mice have greater NF-κB activation and generation of pro-inflammatory cytokines in the brain and spinal cord injury compared to their wild-type Nrf2^+/+^counterparts [78]. Relevant to our study is the fact that chronic TS exposure dampened Nrf2 activity in TBI mice. Thus, the cascading effect of Nrf2 downregulation well fit the slowed recovery, and overall, worse outcomes observed in TBI animals chronically exposed to TS when compared to TBI mice that were not exposed to smoke.

An additional risk factor for TBI patients that is associated with chronic smoking may be derived from the impact of smoking on blood hemostasis. Thrombomodulin is a critical component of the anticoagulant protein C pathway, which ensures control over the process of blood coagulation. This anticoagulant system acts by blocking the activity of the prothrombinase complex and promoting fibrinolysis. This control system is activated following activation of the coagulation cascade in response to vessel injury and/or inflammation [79]. Inflammatory cytokines inhibit thrombomodulin activity, thus blocking NF-kB activation can effectively prevent cytokine-induced downregulation of this anticoagulant factor. Mediated by its pro-inflammatory activity, TS exposure has been previously shown to promote thrombomodulin downregulation and to increase the risk of blood clot formation and stroke [30]. While this may seem of potential benefit for TBI patients, whereas a significant risk of mild TBI is hemorrhage, impairment of blood coagulation related to the inability to control it would be a very undesirable effect. Thrombomodulin is essential to block the activity of the prothrombinase complex and therefore arrest the coagulation cascade. Reduced activity or production of this anticoagulant factor would lead to an uncontrolled coagulation cascade that could extend beyond the damaged area, affecting other (undamaged) vascular districts and restricting local blood flow with consequent ischemic damages and more damage to the blood vessels.

A shortcoming of this study is the exclusive use of male mice rather than a mixed-gender population of animals. Given the “early stage” nature of this study assessing the impact of TS on TBI damage and post-TBI recovery, we felt that it was necessary to minimize the variability of physiological responses (including behavioral) caused by gender-related hormonal differences. However, recent previous studies by our group have highlighted possible gender-specific cellular responses to chronic TS exposure [26] affecting the expression levels of phase 2 enzymes as well as the iron transporters. Although the physiological implications of these differential responses to TS are not clear, they do suggest that gender is a potential risk factor and needs to be investigated. Therefore, we plan to use a mixed gender animal population in follow up studies to determine further pieces of evidence (if any) of a gender-specific impact on TS on TBI and post-TBI recovery. Another limitation of the study is that we have only two sampling points, at 24 and 72 h. This experimental choice, unfortunately, limits the ability to profile the inflammatory activity and antioxidative responses post-injury since a comparison can be made only in respect of the treatments and only between the sampling window.

Although outside the scope of this work, future experiments will focus on further detailing the concurrent underlying mechanisms through which TS impacts TBI. We will also focus on assessing the impact of gender as an additional risk factor in the TS-TBI interplay and expand further to evaluate the effects of vaping on TBI. Targeting Nrf2 to prevent TBI exacerbation by TS and other vascular comorbidities could provide a possible path toward the development of more effective treatments. This latter is of paramount importance for patients that either cannot quit smoking or those that recently stopped but will remain at high risk of developing CNS disorders for a significant amount of time (years) before full renormalization. Crucial consideration must be extended to several other factors that may hinder this renormalization process, including number and type of cigarette smoked per day, years spent smoking, and age of the subject when entirely quit. It is also possible that gender may play a significant role as well.

Although the therapeutic window of BBB regulation after TBI remains unknown, further understanding of the dynamics regulating BBB dysfunction post-TBI would provide essential data to support the development of novel therapeutic approaches, including more selective therapeutic agents, and timing of treatment.

## Conclusion

Chronic cigarette smoking is a modifiable health risk, representing the third leading cause of preventable mortality in the USA [3]. In this study, the effect of TS on the exacerbation of TBI and retardation of post TBI recovery was investigated using a weight-drop model. Considering all the data presented, we showed that TS promotes TBI exacerbation of cerebrovascular injuries and impairs post-TBI healing. Our data suggest that the effects of premorbid TS are consequential to the abrogation of physiological antioxidative and anti-inflammatory response to TBI worsening impairments of the BBB, OS damage, and inflammation. The overall result is that premorbid TS may exacerbate the risk of post-traumatic brain damage and slower recovery.

## Data Availability

The datasets included in this study are available from the corresponding author upon reasonable request.

## Abbreviations

ARE: Antioxidant response element
BBB: Blood-brain barrier
CDC: Centers for Disease Control and Prevention
CHI: Closed head injury
CNS: Central nervous system
CS: Cigarette smoke
2DM: Type 2 diabetes mellitus
FTC: Federal Trade Control
GSH: Glutathione
GSSG: Glutathione disulfide
HO-1: Heme oxygenase 1
ISO: International Organization for Standardization
Keap1: Kelch-like ECH-associated protein 1
NF-ĸB: Nuclear factor kappa-light chain-enhancer of activated B cells
NQO-1: NAD(P)H: quinone reductase I
Nrf2: Nuclear factor erythroid 2-related factor
OS: Oxidative stress
PECAM-1: Platelet endothelial cell adhesion molecule-1
ROS: Reactive oxygen species
SCSM: Single cigarette smoking machine
TBI: Traumatic brain injury
TJ: Tight junction
TS: Tobacco smoke
ZO-1: Zonulae occludentes-1

## References

[1] Laker SR (2011). Epidemiology of concussion and mild traumatic brain injury. PM&R..

[2] Zhang L, Wang H, Fan Y, Gao Y, Li X, Hu Z (2017). Fucoxanthin provides neuroprotection in models of traumatic brain injury via the Nrf2-ARE and Nrf2-autophagy pathways. Scientific reports..

[3] Durazzo TC, Abadjian L, Kincaid A, Bilovsky-Muniz T, Boreta L, Gauger GE (2013). The influence of chronic cigarette smoking on neurocognitive recovery after mild traumatic brain injury. Journal of neurotrauma..

[4] Benady A, Freidin D, Pick CG, Rubovitch V (2018). GM1 ganglioside prevents axonal regeneration inhibition and cognitive deficits in a mouse model of traumatic brain injury. Scientific reports..

[5] McAllister TW (2011). Neurobiological consequences of traumatic brain injury. Dialogues in clinical neuroscience..

[6] Hasan A, Deeb G, Rahal R, Atwi K, Mondello S, Marei HE (2017). Mesenchymal stem cells in the treatment of traumatic brain injury. Front Neurol..

[7] Semple BD, Zamani A, Rayner G, Shultz SR, Jones NC (2019). Affective, neurocognitive and psychosocial disorders associated with traumatic brain injury and post-traumatic epilepsy. Neurobiology of disease..

[8] Sharma R, Shultz SR, Robinson MJ, et al. Infections after a traumatic brain injury: The complex interplay between the immune and neurological systems. Brain Behav Immun. 2019;79:63–74. 10.1016/j.bbi.2019.04.034. 10.1016/j.bbi.2019.04.034 31029794

[9] Rubovitch V, Ten-Bosch M, Zohar O, Harrison CR, Tempel-Brami C, Stein E (2011). A mouse model of blast-induced mild traumatic brain injury. Experimental neurology..

[10] Sivandzade F, Prasad S, Bhalerao A, Cucullo L. Nrf2 and nf-қb interplay in cerebrovascular and neurodegenerative disorders: Molecular mechanisms and possible therapeutic approaches. Redox Biol. 2018. 10.1016/j.redox.2018.11.017 PMC6302038 30576920

[11] Dong W, Yang B, Wang L, Li B, Guo X, Zhang M (2018). Curcumin plays neuroprotective roles against traumatic brain injury partly via Nrf2 signaling. Toxicology and applied pharmacology..

[12] Angeloni C, Prata C, Vieceli Dalla Sega F, Piperno R, Hrelia S. Traumatic brain injury and NADPH oxidase: a deep relationship. Oxidative medicine and cellular longevity. 2015;2015. 10.1155/2015/370312 PMC4397034 25918580

[13] Cornelius C, Crupi R, Calabrese V, Graziano A, Milone P, Pennisi G (2013). Traumatic brain injury: oxidative stress and neuroprotection. Antioxidants & redox signaling..

[14] Ding K, Wang H, Xu J, Li T, Zhang L, Ding Y (2014). Melatonin stimulates antioxidant enzymes and reduces oxidative stress in experimental traumatic brain injury: the Nrf2–ARE signaling pathway as a potential mechanism. Free Radical Biology and Medicine..

[15] Smith JA, Park S, Krause JS, Banik NL (2013). Oxidative stress, DNA damage, and the telomeric complex as therapeutic targets in acute neurodegeneration. Neurochemistry international..

[16] Sivandzade F, Bhalerao A, Cucullo L (2019). Cerebrovascular and neurological disorders: protective role of NRF2. International journal of molecular sciences..

[17] Alves JL (2014). Blood–brain barrier and traumatic brain injury. Journal of neuroscience research..

[18] Chrissobolis S, Miller AA, Drummond GR, Kemp-Harper BK, Sobey CG (2011). Oxidative stress and endothelial dysfunction in cerebrovascular disease. Front Bioscience (Landmark edition).

[19] Ladak AA, Enam SA, Ibrahim MT. A review of the molecular mechanisms of Traumatic Brain Injury. World neurosurgery. 2019. 10.1016/j.wneu.2019.07.039 31301445

[20] Mendes Arent A, Souza LFd, Walz R, Dafre AL. Perspectives on molecular biomarkers of oxidative stress and antioxidant strategies in traumatic brain injury. BioMed research international. 2014;2014. 10.1155/2014/723060 PMC3943200 24689052

[21] Sivandzade F, Cucullo L (2019). Anti-diabetic countermeasures against tobacco smoke-dependent cerebrovascular toxicity: use and effect of rosiglitazone. International journal of molecular sciences..

[22] Sivandzade F, Cucullo L (2019). Assessing the protective effect of rosiglitazone against electronic cigarette/tobacco smoke-induced blood–brain barrier impairment. BMC neuroscience..

[23] Paulson JR, Yang T, Selvaraj PK, Mdzinarishvili A, Van der Schyf CJ, Klein J (2010). Nicotine exacerbates brain edema during in vitro and in vivo focal ischemic conditions. Journal of pharmacology and experimental therapeutics..

[24] Cojocaru IM, Cojocaru M, Sapira V, Ionescu A (2013). Evaluation of oxidative stress in patients with acute ischemic stroke. Romanian journal of internal medicine=. Rev Roum Med Intern.

[25] Cataldo JK, Prochaska JJ, Glantz SA (2010). Cigarette smoking is a risk factor for Alzheimer's disease: an analysis controlling for tobacco industry affiliation. Journal of Alzheimer's disease..

[26] Kaisar MA, Sivandzade F, Bhalerao A, Cucullo L (2018). Conventional and electronic cigarettes dysregulate the expression of iron transporters and detoxifying enzymes at the brain vascular endothelium: In vivo evidence of a gender-specific cellular response to chronic cigarette smoke exposure. Neuroscience letters..

[27] Mutinati M, Pantaleo M, Roncetti M, Piccinno M, Rizzo A, Sciorsci R (2014). Oxidative stress in neonatology. A review. Reproduction in domestic animals..

[28] Wang J-W, Wang H-D, Cong Z-X, Zhou X-M, Xu J-G, Jia Y (2014). Puerarin ameliorates oxidative stress in a rodent model of traumatic brain injury. Journal of Surgical Research..

[29] Prasad S, Sajja RK, Kaisar MA, Park JH, Villalba H, Liles T (2017). Role of Nrf2 and protective effects of Metformin against tobacco smoke-induced cerebrovascular toxicity. Redox biology..

[30] Kaisar MA, Villalba H, Prasad S, Liles T, Sifat AE, Sajja RK (2017). Offsetting the impact of smoking and e-cigarette vaping on the cerebrovascular system and stroke injury: Is Metformin a viable countermeasure?. Redox Biol..

[31] Naik P, Fofaria N, Prasad S, Sajja RK, Weksler B, Couraud P-O (2014). Oxidative and pro-inflammatory impact of regular and denicotinized cigarettes on blood brain barrier endothelial cells: is smoking reduced or nicotine-free products really safe?. BMC neuroscience..

[32] Zhao Y, Luo P, Guo Q, Li S, Zhang L, Zhao M (2012). Interactions between SIRT1 and MAPK/ERK regulate neuronal apoptosis induced by traumatic brain injury in vitro and in vivo. Experimental neurology..

[33] Flierl MA, Stahel PF, Beauchamp KM, Morgan SJ, Smith WR, Shohami E (2009). Mouse closed head injury model induced by a weight-drop device. Nat Protoc..

[34] Kostich W, Hamman BD, Li Y-W, Naidu S, Dandapani K, Feng J, et al. Inhibition of AAK1 kinase as a novel therapeutic approach to treat neuropathic pain. J Pharmacol Exp Ther. 2016:jpet.116.235333. 10.1124/jpet.116.235333 PMC4998676 27411717

[35] Diehl KH, Hull R, Morton D, Pfister R, Rabemampianina Y, Smith D (2001). A good practice guide to the administration of substances and removal of blood, including routes and volumes. J Appl Toxicol Int J..

[36] Adeghe A-H, Cohen J (1986). A better method for terminal bleeding of mice. Lab Anim..

[37] Prasad S, Sajja RK, Park JH, Naik P, Kaisar MA, Cucullo L (2015). Impact of cigarette smoke extract and hyperglycemic conditions on blood–brain barrier endothelial cells. Fluids and Barriers of the CNS..

[38] Sajja RK, Green KN, Cucullo L (2015). Altered nrf2 signaling mediates hypoglycemia-induced blood–brain barrier endothelial dysfunction in vitro. PLoS One..

[39] Ma Q, He X (2012). Molecular basis of electrophilic and oxidative defense: promises and perils of Nrf2. Pharmacological reviews..

[40] Salminen A, Kaarniranta K, Haapasalo A, Hiltunen M, Soininen H, Alafuzoff I (2012). Emerging role of p62/sequestosome-1 in the pathogenesis of Alzheimer's disease. Progress in neurobiology..

[41] Sandberg M, Patil J, D'Angelo B, Weber SG, Mallard C (2014). NRF2-regulation in brain health and disease: implication of cerebral inflammation. Neuropharmacology..

[42] Lozano D, Gonzales-Portillo GS, Acosta S, de la Pena I, Tajiri N, Kaneko Y (2015). Neuroinflammatory responses to traumatic brain injury: etiology, clinical consequences, and therapeutic opportunities. Neuropsychiatr Dis Treat..

[43] Acosta SA, Tajiri N, de la Pena I, Bastawrous M, Sanberg PR, Kaneko Y (2015). Alpha-synuclein as a pathological link between chronic traumatic brain injury and Parkinson's disease. Journal of cellular physiology..

[44] Ozga JE, Povroznik JM, Engler-Chiurazzi EB, Haar CV (2018). Executive (dys) function after traumatic brain injury: special considerations for behavioral pharmacology. Behavioural pharmacology..

[45] Shin SS, Bray ER, Dixon CE (2012). Effects of nicotine administration on striatal dopamine signaling after traumatic brain injury in rats. Journal of neurotrauma..

[46] Chen Y-H, Kuo T-T, Huang EY-K, Hoffer BJ, Kao J-H, Chou Y-C (2018). Nicotine-induced conditional place preference is affected by head injury: correlation with dopamine release in the nucleus accumbens shell. International Journal of Neuropsychopharmacology..

[47] Ilie G, Adlaf EM, Mann RE, Ialomiteanu A, Hamilton H, Rehm J (2015). Associations between a history of traumatic brain injuries and current cigarette smoking, substance use, and elevated psychological distress in a population sample of Canadian adults. Journal of neurotrauma..

[48] Caplan B, Bogner J, Brenner L, Ilie G, Mann RE, Hamilton H (2015). Substance use and related harms among adolescents with and without traumatic brain injury. J Head Trauma Rehabilitation..

[49] Chen Y-H, Kuo T-T, Huang EY-K, Chou Y-C, Chiang Y-H, Hoffer BJ (2018). Effect of traumatic brain injury on nicotine-induced modulation of dopamine release in the striatum and nucleus accumbens shell. Oncotarget..

[50] Freeman LR, Keller JN (2012). Oxidative stress and cerebral endothelial cells: regulation of the blood–brain-barrier and antioxidant based interventions. Biochimica et Biophysica Acta (BBA)-Molecular Basis of Disease.

[51] Villeneuve NF, Lau A, Zhang DD (2010). Regulation of the Nrf2–Keap1 antioxidant response by the ubiquitin proteasome system: an insight into cullin-ring ubiquitin ligases. Antioxidants & redox signaling..

[52] Wang X, Wang Z, Liu JZ, Hu JX, Chen HL, Li WL (2011). Double antioxidant activities of rosiglitazone against high glucose-induced oxidative stress in hepatocyte. Toxicology in Vitro..

[53] Sajja RK, Kaisar MA, Vijay V, Desai VG, Prasad S, Cucullo L (2018). In vitro modulation of redox and metabolism interplay at the brain vascular endothelium: genomic and proteomic profiles of sulforaphane activity. Sci Rep..

[54] Tufekci KU, Civi Bayin E, Genc S, Genc K. The Nrf2/ARE pathway: a promising target to counteract mitochondrial dysfunction in Parkinson's disease. Park Dis. 2011;2011. 10.4061/2011/314082 PMC3049335 21403858

[55] Lu X-Y, Wang H-D, Xu J-G, Ding K, Li T (2015). Deletion of Nrf2 exacerbates oxidative stress after traumatic brain injury in mice. Cellular and molecular neurobiology..

[56] He Y, Yan H, Ni H, Liang W, Jin W (2019). Expression of nuclear factor erythroid 2-related factor 2 following traumatic brain injury in the human brain. NeuroReport..

[57] Zhou Y, Tian M, Wang H-D, Gao C-C, Zhu L, Lin Y-X, et al. Activation of the Nrf2-ARE signal pathway after blast induced traumatic brain injury in mice. Int J Neuroscience. 2019:1–7. 10.1080/00207454.2019.1569652 30648894

[58] Li F, Wang X, Zhang Z, Zhang X, Gao P (2019). Dexmedetomidine attenuates neuroinflammatory–Induced apoptosis after traumatic brain injury via Nrf2 signaling pathway. Annals of clinical and translational neurology..

[59] Sajja RK, Prasad S, Tang S, Kaisar MA, Cucullo L (2017). Blood-brain barrier disruption in diabetic mice is linked to Nrf2 signaling deficits: role of ABCB10?. Neurosci Lett..

[60] Mazzone P, Tierney W, Hossain M, Puvenna V, Janigro D, Cucullo L (2010). Pathophysiological impact of cigarette smoke exposure on the cerebrovascular system with a focus on the blood-brain barrier: expanding the awareness of smoking toxicity in an underappreciated area. International journal of environmental research and public health..

[61] Sivandzade F, Cucullo L (2018). In-vitro blood–brain barrier modeling: a review of modern and fast-advancing technologies. J Cerebral Blood Flow Metab..

[62] Abbott NJ, Patabendige AA, Dolman DE, Yusof SR, Begley DJ (2010). Structure and function of the blood–brain barrier. Neurobiology Dis..

[63] Tomkins O, Feintuch A, Benifla M, Cohen A, Friedman A, Shelef I. Blood-brain barrier breakdown following traumatic brain injury: a possible role in posttraumatic epilepsy. Cardiovascular psychiatry and neurology. 2011;2011. 10.1155/2011/765923 PMC3056210 21436875

[64] Chodobski A, Zink BJ, Szmydynger-Chodobska J (2011). Blood–brain barrier pathophysiology in traumatic brain injury. Translational stroke research..

[65] Ding K, Wang H, Xu J, Lu X, Zhang L, Zhu L (2014). Melatonin reduced microglial activation and alleviated neuroinflammation induced neuron degeneration in experimental traumatic brain injury: possible involvement of mTOR pathway. Neurochemistry international..

[66] Wu L, Chung JY, Saith S, Tozzi L, Buckley EM, Sanders B, et al. Repetitive head injury in adolescent mice: a role for vascular inflammation. J Cerebral Blood Flow Metab. 2018:0271678X18786633. 10.1177/0271678X18786633 PMC6827111 30001646

[67] Acosta SA, Tajiri N, Shinozuka K, Ishikawa H, Grimmig B, Diamond D (2013). Long-term upregulation of inflammation and suppression of cell proliferation in the brain of adult rats exposed to traumatic brain injury using the controlled cortical impact model. PloS one..

[68] Thal SC, Neuhaus W (2014). The blood–brain barrier as a target in traumatic brain injury treatment. Archives of medical research..

[69] Shlosberg D, Benifla M, Kaufer D, Friedman A (2010). Blood–brain barrier breakdown as a therapeutic target in traumatic brain injury. Nature Reviews Neurology..

[70] Ye L, Huang Y, Zhao L, Li Y, Sun L, Zhou Y (2013). IL-1β and TNF-α induce neurotoxicity through glutamate production: a potential role for neuronal glutaminase. Journal of neurochemistry..

[71] Carvalho C, Moreira PI (2018). Oxidative stress: a major player in cerebrovascular alterations associated to neurodegenerative events. Front Physiol.

[72] Popescu BO. Triggers and effectors of oxidative stress at blood-brain barrier level: relevance for brain ageing and neurodegeneration. Oxid Med Cell Longev. 2013;2013. 10.1155/2013/297512 PMC3606793 23533687

[73] Lochhead JJ, McCaffrey G, Quigley CE, Finch J, DeMarco KM, Nametz N (2010). Oxidative stress increases blood–brain barrier permeability and induces alterations in occludin during hypoxia–reoxygenation. J Cerebral Blood Flow Metab..

[74] Liu Z-M, Chen Q-X, Chen Z-B, Tian D-F, Li M-C, Wang J-M (2018). RIP3 deficiency protects against traumatic brain injury (TBI) through suppressing oxidative stress, inflammation and apoptosis: Dependent on AMPK pathway. Biochemical and biophysical research communications..

[75] Mettang M, Reichel SN, Lattke M, Palmer A, Abaei A, Rasche V (2018). IKK2/NF-κB signaling protects neurons after traumatic brain injury. The FASEB Journal..

[76] Khan NM, Haqqi TM (2017). Pleiotropic roles of Nrf2 as regulators of chondrocyte apoptosis, oxidative stress, inflammatory response and catabolic and anabolic pathways in osteoarthritis. Free Radical Biology and Medicine..

[77] Niture SK, Jaiswal AK (2013). Nrf2-induced antiapoptotic Bcl-xL protein enhances cell survival and drug resistance. Free Radical Biology and Medicine..

[78] Mao L, Wang H, Qiao L, Wang X. Disruption of Nrf2 enhances the upregulation of nuclear factor-kappaB activity, tumor necrosis factor-, and matrix metalloproteinase-9 after spinal cord injury in mice. Mediators of inflammation. 2010;2010. 10.1155/2010/238321 PMC2938451 20862369

[79] Conway EM, editor Thrombomodulin and its role in inflammation. Seminars in immunopathology; 2012: Springer. 10.1007/s00281-011-0282-8 21805323

